# Challenges and clinical relevance of molecular detection of *Bordetella pertussis* in South Africa

**DOI:** 10.1186/s12879-019-3869-7

**Published:** 2019-03-21

**Authors:** Fahima Moosa, Mignon du Plessis, Nicole Wolter, Maimuna Carrim, Cheryl Cohen, Claire von Mollendorf, Sibongile Walaza, Stefano Tempia, Halima Dawood, Ebrahim Variava, Anne von Gottberg

**Affiliations:** 1 Centre for Respiratory Diseases and Meningitis, National Institute for Communicable Diseases of the National Health Laboratory Service, Private Bag X4, Sandringham, Gauteng 2131 South Africa; 2 University of the Witwatersrand, Johannesburg, South Africa; 3 Centers for Disease Control and Prevention, Atlanta, GA USA; 4 Pietermaritzburg Metropolitan Hospitals, KwaZulu-Natal, and Caprisa, University of KwaZulu-Natal, Durban, South Africa; 5 Klerksdorp-Tshepong Hospital, North West Province, Klerksdorp, South Africa

**Keywords:** *Bordetella pertussis*, South Africa, Severe respiratory illness, Influenza-like illness (ILI), Surveillance, Real-time PCR, Confirmed-pertussis, Possible-pertussis, Attributable fraction

## Abstract

**Background:**

We assessed the utility of a multi-target, real-time PCR assay for *Bordetella pertussis* detection and diagnosis in patients with severe respiratory illness (SRI), influenza-like illness (ILI), and asymptomatic controls.

**Methods:**

Real-time PCR detection of IS*481*, pIS*1001*, hIS*1001* and *ptxS1* was performed on nasopharyngeal specimens (SRI, ILI and controls) and induced sputum (SRI) collected from June 2012 to May 2016 through respiratory illness surveillance. Using PCR cycle threshold (C_t_) value cut-offs, IS*481* positive cases were classified as confirmed (C_t_ < 35) or possible (C_t_ 35–39) pertussis disease.

**Results:**

Among 12,922 samples, 146 (1.1%) were IS*481* positive of which 62% (90/146) were classified as confirmed. The attributable fraction (AF) was 92.2% (95% CI, 65.6 to 98.2%) and 90.5% (95% CI, 57.5 to 97.9%) amongst SRI and ILI PCR-confirmed pertussis cases, respectively. Amongst possible pertussis cases, AF was 36.9% (95% CI, − 142.3 to 83.6%) and 67.5% (95% CI, − 30.6 to 91.9%) in the SRI and ILI groups, respectively.

**Conclusion:**

All IS*481* positive specimens could be considered as *B. pertussis* infection, and potentially pertussis disease with supportive clinical information.

**Electronic supplementary material:**

The online version of this article (10.1186/s12879-019-3869-7) contains supplementary material, which is available to authorized users.

## Background

Pertussis (whooping cough) is caused by the vaccine-preventable, bacterial pathogen *Bordetella pertussis* [1, 2]. Despite high vaccine coverage with the whole-cell or acellular vaccines and an initial substantial decrease following vaccine introduction, the incidence of pertussis has increased globally during the last two decades [3–7]. This has been attributed to increased awareness by clinicians, more sensitive molecular techniques for diagnosis [4, 7], serological markers for identification of infection in adolescents and adults who usually present atypically with *B*. *pertussis* [3], pathogen adaptation and/or waning vaccine immunity [6, 8].

The changing epidemiology of pertussis disease requires new control strategies but establishing the burden of pertussis disease in low-resource settings is challenging due to the absence of effective surveillance systems. As such, pertussis surveillance can potentially be linked to surveillance for other respiratory illnesses, such as influenza or pneumonia surveillance [9]. Differences in case definitions, however, may result in reduced sensitivity for detecting pertussis cases using these platforms.

Different laboratory methods such as bacterial culture, molecular testing and serology may be used to confirm a clinical diagnosis of pertussis, however, the sensitivity of each method is dependent on the stage of disease and the age of the individual. Culture and molecular testing are recommended for diagnosis during the early (catarrhal and paroxysmal) phases of disease, whereas serology is recommended during the convalescent phase [10, 11]. The sensitivity of both culture and PCR appear to be highest in young infants and decline in adolescents and adults [12].

Real-time PCR detection of *B. pertussis* is limited by a lack of validated and specific gene targets. The most commonly used PCR target is the multi-copy insertion sequence gene IS*481* (present in 50–250 copies per genome); however, this gene is also found in *B*. *holmesii* and *B*. *bronchiseptica* [13, 14]. Use of a second confirmatory target such as the pertussis toxin subunit gene (*ptxS1)* improves specificity of *B. pertussis* detection [7, 15, 16].

The high copy number of IS*481* enables highly sensitive detection of *B. pertussis*, however, this results in uncertainty regarding the interpretation of results with high PCR cycle threshold (C_t_) values that cannot always be confirmed by detection of a less sensitive, single copy *ptxS1* assay. Multiple copies of IS*481* increase the risk of laboratory contamination and detection of false positives [17, 18]. Bacterial load in the host and resulting C_t_ values may be influenced by time between symptom onset and specimen collection, immune status of the host, prior use of antimicrobial therapy or quality of the sample collected [19–21]. The interpretation and clinical relevance of high C_t_ values, in a setting where stringent laboratory control measures are available to prevent and detect IS*481* contamination, is not clearly understood.

We aimed to evaluate a multi-target real-time PCR assay and the use of C_t_ value cut-offs for *B. pertussis* detection and diagnosis in patients with mild or severe respiratory illness at two sentinel sites in South Africa.

## Methods

### Surveillance population

We enrolled patients and asymptomatic controls through two surveillance systems (one for severe respiratory illness (SRI) and one for influenza-like illness (ILI)) from June 2012 through May 2016. Patients of all ages, hospitalised with lower respiratory tract infection, irrespective of symptom duration, were enrolled in prospective, active surveillance as part of severe respiratory illness (SRI) surveillance at Edendale Hospital and Klerksdorp-Tshepong Hospital Complex (KTHC) [22]. From August 2014, case investigation forms used for patient enrolment were amended to capture clinical symptoms consistent with pertussis disease, namely, paroxysms of cough, inspiratory whoop, apnea (with or without cyanosis) and posttussive vomiting. Patients with influenza-like illness (ILI), and asymptomatic controls were enrolled at the respective outpatient clinics affiliated to each hospital [Edendale Gateway and Jouberton clinics [23]]. A case of ILI was defined as an outpatient presenting with acute fever (> 38 °C) and/or self-reported fever and cough or sore throat within the last 10 days. A control was defined as a person presenting at the same outpatient clinic with no history of fever, respiratory or gastrointestinal symptoms during the 14 days preceding the visit. Controls were not followed-up for development of respiratory symptoms after enrolment. We aimed to enrol one HIV-infected and one HIV-uninfected control every week in each clinic within the following age categories: 0–1, 2–4, 5–14, 15–54 and ≥ 55 years (controls were enrolled by HIV status so that the study was powered sufficiently for HIV-stratified analysis). Demographic and clinical data were collected by surveillance officers through interview and hospital record review. For children aged < 5 years, immunization history (including pertussis immunization) was documented and confirmed from the vaccination card if available.

### Specimen collection and processing

Nasopharyngeal (NP) specimens were collected from patients and controls as follows: NP aspirates were collected from children (< 5 years), whereas both NP and oropharyngeal (OP) swabs were collected from older individuals (≥5 years) and stored together in a single vial of a 3 ml solution of Universal Transport Medium (UTM) (Copan, Italia, Brescia, Italy). Induced sputum was collected from hospitalised (SRI) patients only. NPs were stored at 4 °C and transported on ice packs and induced sputum was stored at − 20 °C and transported on dry ice to the National Institute for Communicable Diseases (NICD) in Johannesburg for testing. From August 2014, all SRI- and ILI-enrolled individuals with pertussis symptoms had a second NP collected and inoculated into Regan-Lowe medium (Media Mage, Johannesburg, South Africa) for culture at the NICD. Induced sputum and Regan-Lowe swabs were cultured on charcoal agar (Diagnostic Media Products, Johannesburg, South Africa)*.* Plates were incubated at 37 °C for seven days and inspected for growth on days three and seven. Suspected *B. pertussis* colonies were confirmed using matrix-assisted laser desorption/ionization time of flight mass spectrometry (MALDI-TOF, Bruker, Massachusetts, United States) and by real-time PCR detection of IS*481* and *ptxS1* genes [16].

### Detection of *Bordetella* species by real-time PCR

Total nucleic acids were extracted from 200 μl of UTM or digested induced sputum using a Roche MagNa Pure 96 instrument (Roche Diagnostics, Mannheim, Germany) and MagNa Pure 96 DNA and Viral NA SV Kit (Roche Diagnostics) using the Pathogen Universal protocol. Real-time PCR was performed using an Applied Biosystems 7500 Fast instrument (Applied Biosystems, Foster City, United States) targeting IS*481*, pIS*1001*, hIS*1001* and *ptxS*1 as previously described [16]. The targets differentiate *B*. *pertussis*, *B*. *parapertussis*, *B*. *holmesii* and *B*. *bronchiseptica*. PCR assays were performed in 96-well plates including 16 negative extraction controls and 16 no-template controls distributed throughout the plate to detect IS*481* contamination. An internal validation was performed for the assay and 100% sensitivity and specificity was obtained for the detection of *B. parapertussis*, *B. bronchiseptica* and *B. holmesii*; and 95% sensitivity and 100% specificity was obtained for the detection of *B. pertussis*. All testing was conducted at the NICD, at an accredited reference laboratory using standardised procedures and testing algorithms.

### Real-time PCR result interpretation and PCR quality control

A specimen was considered positive for *B. pertussis* DNA if both IS*481* and *ptxS1* were detected with C_t_ values ≤39. Additionally, samples that were positive for IS*481* only (with C_t_ values 35–39 and without confirmation of *ptxS1*) and negative for pIS*1001* (*B. parapertussis*) or hIS*1001* (*B. holmesii*) were recorded as *B. pertussis* positive. Samples with positive PCR results (IS*481* and/or *ptxS1*) were further categorised as confirmed or possible *B. pertussis* based on IS*481* C_t_ value cut-offs of < 35 and 35–39, respectively. The testing algorithm was as follows: specimens that tested positive for IS*481* on initial screening underwent repeat testing, namely, new extraction, and PCR in duplicate. Results were recorded as positive if IS*481* was detected in at least two replicates, taking into account the result from the primary screen.

Detection of the human ribonuclease P gene (*RNase P*) served as a control to exclude the presence of PCR inhibitors and confirm specimen quality [24]. To exclude spurious amplification, a 1014-bp fragment of the IS*481* gene from a selection of confirmed (*n* = 13) and possible (*n* = 14) *B. pertussis* PCR-positive samples was amplified and sequenced as described previously [16]. Sequences were analysed using DNAStar Lasergene SeqMan Pro software (DNAStar, Wisconsin, United States) and the Basic Local Alignment Search Tool (BLAST).

Higher than expected PCR detection rates of IS*481* (based on baseline data) at any point during the study period were investigated to exclude laboratory and/or environmental contamination: (i) all laboratory processes were audited, (ii) environmental samples were collected from appropriate areas at surveillance sites and laboratory surfaces were swabbed and tested by real-time PCR for IS*481* and (iii) individuals that tested positive for *B*. *pertussis* during this time were retrospectively interviewed using a standardised questionnaire to collect data on pertussis-related symptoms.

### Data analysis

The prevalence of *B*. *pertussis* by patient group (SRI, ILI and controls) and 95% confidence intervals (CI) was calculated. To determine the optimal specimen type for molecular detection of *B*. *pertussis*, the detection rates in NP and induced sputum were compared using the chi-squared test among individuals that had both specimen types collected and tested. We estimated the fraction of *B. pertussis* detection (IS*481*-positive PCR results) attributable (attributable fraction [AF]) to severe (SRI) (including only patients that tested positive on NP specimens) or mild (ILI) illness using unconditional logistic regression by comparing the *B. pertussis* detection rate among SRI or ILI cases to those of controls. All models were adjusted for HIV status and age. The AF was estimated from the odds ratio (OR) obtained from the regression models using the following equation: AF = (OR-1)/(OR) × 100 as previously described [25]. An OR was used for these calculations as the data were analysed considering the case-control design of this study. The AF in this study provides an estimate of the proportion of pertussis-positive patients who have symptomatic illness (either SRI or ILI as defined in this study) resulting from pertussis infection. This analysis was implemented overall as well as among confirmed (NP IS*481* C_t_ < 35) and possible (NP IS*481* C_t_ 35–39) cases. In addition, we compared the demographic and clinical characteristics of possible vs. confirmed IS*481*-positive patients with SRI or ILI using univariate unconditional conventional or penalised (for categorical variables with 0 outcome in one or more categories) logistic regression. For patients with SRI, confirmed and possible cases were compared for (i) patients that tested positive for IS*481* on either one or both specimen types (where both specimen types were available and tested positive for *B*. *pertussis*, the lower of the two C_t_ values was recorded) and (ii) patients that tested positive for IS*481* on NP specimens only. Statistical analyses were performed using Stata® version 14 (Statacorp, Texas, United States). Statistical significance was determined at *p* < 0.05 for all analyses.

### Ethics approval

Ethics for SRI (M081042) and ILI (M120133) surveillance and *B. pertussis* testing were approved by the University of the Witwatersrand (M130260) and the University of KwaZulu Natal (BF081/12), South Africa.

## Results

### Study population

**Fig. 1:**
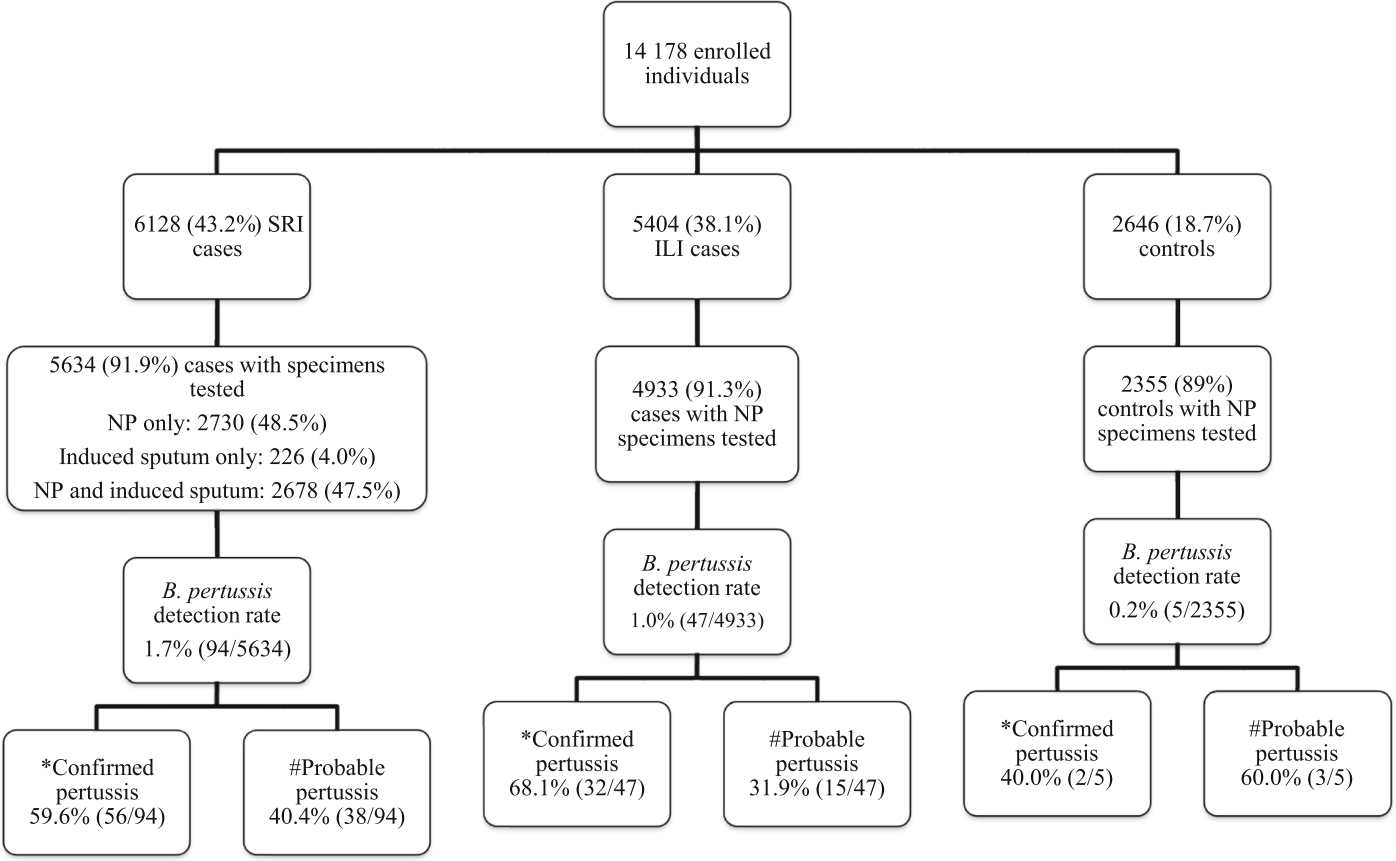
Flow diagram depicting breakdown of individuals enrolled, specimens collected and tested, and *B. pertussis* prevalence for severe respiratory (SRI) and influenza-like illness (ILI) surveillance, and controls, South Africa, June 2012 – May 2016. NP: nasopharyngeal aspirates from children < 5 years of age and combined nasopharyngeal and oropharyngeal swabs from individuals aged ≥5 years. *Confirmed = positive for *B. pertussis* with IS*481* Ct < 35; Possible = positive for *B. pertussis* with IS*481* 35 ≥ Ct ≤ 39

From June 2012 through May 2016, 14,178 individuals were enrolled. Testing was conducted on 12,922 (91%) specimens: 5634 (44%) from SRI patients, 4933 (38%) from ILI patients and 2355 (18%) from control subjects (Fig. 1). Specimens were not tested or available for 9% of enrolled individuals as participants were either too sick to have specimens taken or did not give consent for testing or were discharged before specimens could be collected. Children aged < 5 years accounted for 37% (2071/5618), 33% (1618/4921) and 33% (787/2352) of SRI cases, ILI cases, and controls, respectively (Table 1). The HIV prevalence was 51% (2731/5308) among SRI cases, 27% (1198/4451) among ILI cases and 42% (949/2263) among controls (enrolment criteria for controls was based on HIV status).

**Table 1:** Demographic and clinical characteristics of patients enrolled for severe respiratory and influenza-like illness surveillance and asymptomatic controls, South Africa, June 2012 – May 2016 (*N* = 12,922)

Characteristic	Surveillance population		
	SRI n/N (%) *N* = 5634	ILI n/N (%) *N* = 4933	Controls n/N (%) *N* = 2355
Gender			
Male	2861/5626 (51)	1757/4733 (37)	774/2291 (34)
Female	2765/5626 (49)	2976/4733 (63)	1517/2291 (66)
Race			
Black	5473/5604 (98)	4723/4728 (100)	2287/2292 (100)
Non-black	131/5604 (2)	5/4728 (0)	5/2292 (0)
Age group (years)			
< 1	1307/5618 (23)	599/4921 (12)	323/2352 (14)
1–4	764/5618 (14)	1019/4921 (21)	464/2352 (20)
5–14	168/5618 (3)	727/4921 (15)	520/2352 (22)
15–24	276/5618 (5)	649/4921 (13)	191/2352 (8)
25–44	1756/5618 (31)	1388/4921 (28)	371/2352 (16)
45–64	1051/5618 (19)	460/4921 (9)	337/2352 (14)
≥ 65	296/5618 (5)	79/4921 (2)	146/2352 (6)
Underlying illness^*^			
No	5023/5627 (89)	4484/4723 (95)	2172/2290 (95)
Yes	604/5627 (11)	239/4723 (5)	118/2290 (5)
HIV status			
Uninfected	2577/5308 (49)	3253/4451 (73)	1314/2263 (58)
Infected	2731/5308 (51)	1198/4451 (27)	949/2263 (42)
Hospital/Clinic			
Edendale	2507/5634 (45)	N/A	N/A
KTHC	3127/5634 (56)	N/A	N/A
Edendale Gateway	N/A	3655/4933 (74)	1041/2355 (44)
Jouberton Clinic	N/A	1278/4933 (26)	1314/2355 (56)

SRI = Severe respiratory illness; ILI = Influenza-like illness; N/A = Not applicable; KTHC = Klerksdorp Tshepong hospital complex

^*^Patients with previously diagnosed chronic conditions including asthma, chronic lung diseases, cirrhosis/liver failure, chronic renal failure, heart failure, valvular heart disease, coronary heart disease, immunosuppressive therapy, splenectomy, diabetes, burns, kwashiorkor/marasmus, nephrotic syndrome, spinal cord injury, seizure disorder, emphysema, or cancer. All percentages are rounded off

### PCR detection rate of *B. pertussis*

Overall, *B*. *pertussis* was detected in 1.1% (146/12,922, 95% CI 1.4 to 1.8) of individuals with detection rates of 1.7% (94/5634, 95% CI 2.0 to 2.8), 1.0% (47/4933, 95% CI 0.9 to 1.5) and 0.2% (5/2355, 95% CI 0.1 to 0.6) in the SRI, ILI and control groups, respectively (SRI vs. ILI *p* < 0.0001) (Fig. 1). *B*. *parapertussis* was detected in 0.8% (46/5634), 0.4% (22/4933) and 0.2% (4/2355) of SRI, ILI and control individuals, respectively. We did not detect *B*. *pertussis* and *B*. *parapertussis* co-infections. In addition, neither *B*. *holmesii* nor *B*. *bronchiseptica* was detected.

Among SRI cases with both specimen types collected, the detection rate was 0.2% (6/2678) for NP specimens (using NP specimens only) and 1.2% (31/2678) for induced sputum (using induced sputum only) (*p* < 0.0001). In 0.9% (25/2678) of patients, both specimen types were positive for *B*. *pertussis*. Where both specimen types tested positive for *B. pertussis*, induced sputum yielded lower C_t_ values [mean C_t_ value (±standard deviation) 24 ± 7] compared to NP specimens (mean C_t_ value 31 ± 7) (*p* = 0.002).

Of 3104 induced sputum samples, 3 (0.1%) were culture positive for *B. pertussis*. From August 2014 to May 2016, 0.1% (4/4583) of NPs were culture positive for *B. pertussis*. All isolates were from IS*481*-positive samples with C_t_ values < 35.

### Confirmed and possible pertussis cases

Overall, 146 PCR-positive *B. pertussis* individuals (patients positive on either NP or induced sputum) were identified in all three surveillance groups, of which 62% (90/146) were categorised as confirmed (Fig. 1). The proportion of confirmed cases did not differ significantly by study population (SRI: 60%, 56/94; ILI: 68%, 32/47; controls: 40%, 2/5 (40%); *p* = 0.37).

**Fig. 2:**
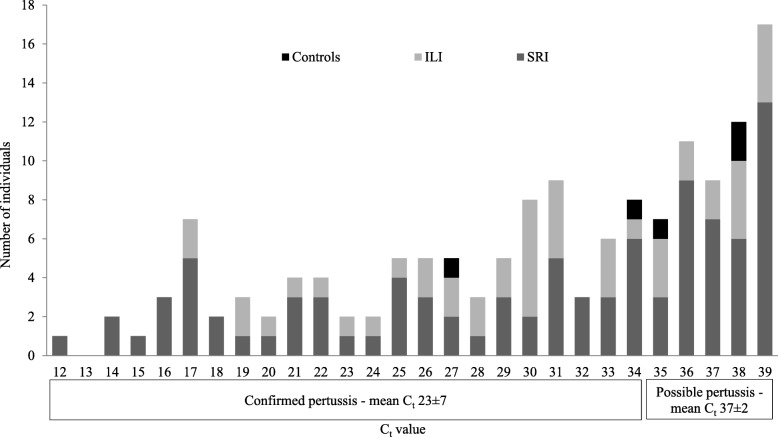
Distribution of real-time PCR IS*481* C_t_ values for confirmed (*n* = 90) and possible (*n* = 56) pertussis cases, by surveillance population, South Africa, June 2012 – May 2016. SRI = severe respiratory illness; ILI = influenza-like illness

The mean C_t_ value was 23 ± 7 for confirmed cases and 37 ± 2 for possible cases (Fig. 2). Of the confirmed and possible cases, 92% (83/90) and 7% (4/56) were also positive for the *ptxS*1 gene, respectively. The sequences of IS*481* amplicons from 12/13 (92%) samples from confirmed and 6/14 (43%) samples from possible cases were confirmed through BLAST analysis. IS*481* amplicons from one confirmed (C_t_ = 34) and eight possible (C_t_ range 35–39) cases could not be confirmed by sequencing although PCR amplicons of the expected size were confirmed using agarose gel electrophoresis.

### *B*. *pertussis* attributable fraction (AF)

For SRI patients compared to controls, the overall AF of *B*. *pertussis* detection to respiratory disease was 72.4% (95% CI, 41.0 to 87.1%); with 92.2% (95% CI, 65.6 to 98.2%) and 36.9% (95% CI, − 142.3 to 83.6%) for confirmed and possible cases, respectively. For ILI cases compared to controls, the overall AF was 77.3% (95% CI, 50.8 to 89.5%); with 90.5% (95% CI, 57.5 to 97.9%) and 67.5% (95% CI, − 30.6 to 91.9%) for confirmed and possible cases, respectively.

### Patient characteristics among pertussis-confirmed and -possible cases

Among SRI patients, 1.7% (94/5634) were PCR positive for *B. pertussis*, of which 60% (56/94) were categorised as confirmed *B. pertussis* cases. Possible cases were more likely to occur among patients aged 45–64 years than among patients aged < 1 year (OR 6.3, 95% CI 1.7 to 23.8) (Table 2). Restricting the analysis to include cases that were positive on NP specimens only, there were no differences between the confirmed and possible cases (Additional file 1). There were no differences in the demographic and clinical characteristics between the confirmed and possible cases that tested positive on either NP and/or sputum specimens or on NP specimens only.

**Table 2:** Comparison of confirmed (*N* = 56) and possible (*N* = 38) pertussis cases (real-time PCR positive for IS*481* in nasopharyngeal or induced sputum specimens) in hospitalised patients with severe respiratory illness, South Africa, June 2012 – May 2016 (*N* = 94)

Characteristic	Confirmed pertussis^*^ n/N^#^ (%)	Possible pertussis^*^ n/N^#^ (%)	OR^†^	*P* value
			(95% CI)	
Year				
2012	7/56 (12.5)	4/38 (10.5)	Reference	
2013	9/56 (16)	9/38 (24)	1.8 (0.4–8.1)	0.48
2014	16/56 (29)	13/38 (34)	1.4 (0.3–5.9)	0.63
2015	22/56 (39)	10/38 (26)	0.8 (0.1–3.4)	0.76
2016	2/56 (4)	2/38 (5)	1.8 (0.2–17.7)	0.64
Gender				
Male	24/56 (43)	21/38 (55)	Reference	
Female	32/56 (57)	17/38 (45)	0.6 (0.3–1.4)	0.24
Age group (years)^¶^				
< 1	23/56 (41)	9/38 (24)	Reference	
1–4	6/56 (11)	5/38 (13)	2.1 (0.5–8.2)	0.29
5–14	5/56 (9)	2/38 (5)	1.1 (0.2–6.0)	0.89
15–24	1/56 (2)	0/38 (0)	0.8 (0.03–22.1)	0.91
25–44	15/56 (27)	11/38 (29)	1.8 (0.6–5.4)	0.27
45–64	4/56 (7)	11/38 (29)	**6.3 (1.7–23.8)**	**0.006**
≥ 65	2/56 (4)	0/38 (0)	0.5 (0.02–11.3)	0.66
Fever history				
No	28/55 (51)	21/38 (55)	Reference	
Yes	27/55 (49)	17/38 (45)	0.8 (0.4–1.9)	0.68
HIV status				
Uninfected	27/53 (51)	15/37 (41)	Reference	
Infected	26/53 (49)	22/37 (59)	1.5 (0.7–3.6)	0.33
HIV treatment				
No	7/22 (32)	9/19 (47)	Reference	
Yes	15/22 (68)	10/19 (53)	0.5 (0.1–1.8)	0.31
Symptom duration				
< 7 days	35/56 (62.5)	23/38 (60.5)	Reference	
7–20 days	10/56 (18)	5/38 (13)	0.8 (0.2–2.5)	0.65
≥ 21 days	11/56 (20)	10/38 (26)	1.4 (0.5–3.8)	0.53
Underlying illness^‡^				
No	50/56 (89)	30/38 (79)	Reference	
Yes	6/56 (11)	8/38 (21)	2.2 (0.7–7.0)	0.17
ICU				
No	53/55 (96)	37/38 (97)	Reference	
Yes	2/55 (4)	1/38 (3)	0.7 (0.06–8.2)	0.79
Antibiotic treatment (24 h)				
No	51/56 (91)	36/38 (95)	Reference	
Yes	5/56 (9)	2/38 (5)	0.6 (0.1–3.1)	0.10
Hospital duration				
< 2 days	5/52 (10)	5/38 (13)	Reference	
2–4 days	18/52 (35)	17/38 (45)	0.9 (0.2–3.9)	0.94
5–7 days	10/52 (19)	5/38 (13)	0.5 (0.1–2.6)	0.41
≥ 8 days	19/52 (36.5)	11/38 (29)	0.6 (0.1–2.5)	0.56
Viral co-infection				
Viral co-infectionNo	32/56 (57)	24/38 (63)	Reference	
Viral co-infectionYes	24/56 (43)	14/38 (37)	0.8 (0.3–1.8)	0.56
Outcome				
Survived	50/54 (93)	37/38 (97)	Reference	
Died	4/54 (7)	1/38 (3)	0.3 (0.04–3.1)	0.34
Vaccination for age^§^				
Full coverage	16/22 (73)	10/14 (71)	Reference	
Incomplete	6/22 (27)	4/14 (29)	0.9 (0.2–4.2)	0.93
Facility				
Edendale	26/56 (46)	12/38 (32)	Reference	
KTHC	30/56 (54)	26/38 (68)	1.9 (0.8–4.4)	0.15

OR = Odds ratio; CI = confidence interval; KTHC = Klerksdorp Tshepong Hospital complex. ^*^Confirmed case = positive for *B*. *pertussis* with IS*481* C_t_ < 35; Possible case = positive for *B*. *pertussis* with IS*481* 35 ≥ C_t_ ≤ 39. ^#^Data unknown/missing for some cases accounting for the different denominators. ^†^Odds ratio calculated for confirmed versus possible pertussis cases using univariate logistic regression. Bold font indicates statistical significance

^‡^Patients with previously diagnosed chronic conditions including asthma, chronic lung diseases, cirrhosis/liver failure, chronic renal failure, heart failure, valvular heart disease, coronary heart disease, immunosuppressive therapy, splenectomy, diabetes, burns, kwashiorkor/marasmus, nephrotic syndrome, spinal cord injury, seizure disorder, emphysema, or cancer. ^§^For children ≤5 years of age where vaccine history was available and documented on vaccination card. All percentages are rounded off. ^¶^ Estimated using penalised logistic regression

Among ILI patients, 1% (47/4933) were PCR positive for *B*. *pertussis*, of which 68% (32/47) were confirmed cases. There was no difference in the demographic and clinical characteristics between confirmed and possible cases in this group (Additional file 2).

### Laboratory and site investigation

In August 2014, there was a higher than usual PCR detection rate for *B*. *pertussis* (4.3%, 16/368) compared to previous months. The increase occurred in samples collected at one geographical site (KTHC and Jouberton Clinic). An evaluation of all laboratory systems and testing of 32 environmental samples collected from the affected hospital and clinic excluded facility and laboratory contamination, indicating a true increase in *B*. *pertussis* infection. In addition, 17 PCR-positive patients [9/17 (53%) SRI and 8/17 (47%) ILI], enrolled during the period when the increase in *B. pertussis* detection was observed, were retrospectively interviewed (Table 3). Eleven patients (65%) met the clinical case definition for pertussis disease, namely, cough for > 2 weeks (any duration in infants < 1 year) + one of the following: paroxysmal cough, inspiratory whoop, posttussive vomiting, apnea among infants < 1 year. Of these, 78% (7/9) and 50% (4/8) were confirmed and possible cases, respectively. No epidemiological links were identified between these individuals.

**Table 3:** Characteristics of individuals with *B*. *pertussis* PCR-positive results that were interviewed following the unusual increase in *B. pertussis* PCR detection rate in August 2014 (*N* = 17)

Case	Age group	HIV status	Confirmed/possible pertussis^*^	Cough ≥2 weeks	Paroxysmal cough	Inspiratory whoop	Posttussive vomiting	Apnea	Meets clinical case definition^†^
1	< 1y	Negative	Possible	No	No	No	No	No	No
2	1-4y	Positive	Confirmed	Yes	Yes	No	No	N/A	Yes
3	25-44y	Positive	Confirmed	No	No	No	No	No	No
4	25-44y	Negative	Confirmed	Yes	Yes	No	No	N/A	Yes
5	25-44y	Positive	Possible	Yes	No	No	No	N/A	No
6	45-64y	Negative	Confirmed	Yes	No	No	No	N/A	No
7	45-64y	Negative	Possible	Yes	No	No	No	N/A	No
8	45-64y	Positive	Possible	Yes	Yes	Yes	No	N/A	Yes
9	1y	Negative	Confirmed	Yes	Yes	No	No	Yes	Yes
10	25-44y	Positive	Confirmed	Yes	Yes	Yes	No	N/A	Yes
11	< 1y	Unknown	Confirmed	Yes	Yes	Yes	Yes	Yes	Yes
12	1-4y	Unknown	Possible	Yes	No	No	No	N/A	No
13	1-4yy	Positive	Possible	Yes	No	No	Yes	N/A	Yes
14	5-14y	Unknown	Possible	Yes	Yes	No	No	N/A	Yes
15	25-44y	Negative	Possible	Yes	No	No	Yes	N/A	Yes
16	25-44y	Negative	Confirmed	Yes	Yes	Yes	No	N/A	Yes
17	5-14y	Unknown	Confirmed	Yes	Yes	Yes	Yes	N/A	Yes

y = age in years. ^*^Confirmed case = positive for *B*. *pertussis* with IS*481* C_t_ < 35; Possible case = positive for *B*. *pertussis* with IS*481* 35 ≥ C_t_ ≤ 39. ^†^Clinical case definition = cough > 2 weeks (any duration in infants <1 year) + one of the following: paroxysmal cough, inspiratory whoop, posttussive vomiting, apnea in infants < 1 year

## Discussion

In the absence of pertussis-specific surveillance in South Africa, we evaluated the utility of real-time PCR detection of *B. pertussis* using an existing SRI and ILI surveillance platform. In our setting, laboratory methods were standardised and *B. pertussis* laboratory contamination could be excluded. As such, all individuals in whom IS*481* was detected and *B. holmesii*, *B. parapertussis* and *B. bronchiseptica* were excluded, were considered to have *B. pertussis* DNA in their respiratory tract at the time of sampling and were therefore considered positive for *B. pertussis* infection. *B*. *bronchiseptica* has been associated with HIV-infected individuals [26–29]; however, this pathogen was not detected in our study. In confirmed cases, the attributable fraction of *B. pertussis* detection to respiratory disease was significant for both hospitalised (SRI) and mild (ILI) cases. However, for PCR-possible cases (both SRI and ILI) there was no significant association with *B*. *pertussis* disease.

In individuals with high C_t_ values (> 35), low bacterial loads may represent asymptomatic colonisation, or residual DNA post disease, or pertussis disease [19, 30]. Therefore, the use of stringent C_t_ value cut-offs may still result in the misdiagnosis of some true pertussis cases given that adults and adolescents have been shown to harbor lower bacterial loads and may present with milder disease or atypical symptoms [19, 30, 31]. In our study, just over one-third of *B. pertussis* PCR-positive cases had low bacterial loads (C_t_ > 35) and were defined as possible pertussis cases. This is potentially due to the case definition that was not specific for pertussis and the enrollment of individuals of all ages. We also showed that in hospitalised patients, pertussis-possible individuals were more likely to be adults (45–64 years of age) than infants (< 1 year of age) compared with pertussis-confirmed individuals.

In our study, a subset of 17 patients that were PCR positive for *B*. *pertussis* (confirmed and possible cases) from 2014 were retrospectively interviewed. The majority of confirmed cases reported clinical symptoms consistent with pertussis; however, 50% (4/8) of possible cases also had clinical pertussis. Since this was only a small group of individuals, we are prospectively conducting active case follow-up to understand the association between PCR C_t_ values and pertussis symptoms. In Canada, Public Health Ontario Laboratories determined the association between PCR C_t_ with pertussis severity and symptoms, and showed that the proportion of patients with pertussis symptoms did not differ between individuals with low (C_t_ < 36) and high IS*481* C_t_ values [19].

IS*481* is a multi-copy target with up to 250 copies per *B. pertussis* genome which increases assay sensitivity, but also increases the risk of false positives due to environmental or laboratory contamination [17, 18, 32]. As such, different C_t_ value cut-offs have been recommended when interpreting PCR data and these vary between studies. In a Tunisian study, *B*. *pertussis* cases were defined as PCR positive for *IS481* and *ptxS1* with a C_t_ < 45, or as *Bordetella* spp. if they were positive for *IS481* only with a C_t_ < 45 [7]. The assay currently used by our laboratory detects one genomic equivalent of *B. pertussis* at an average C_t_ of 33.3 [16]. This, together with variable PCR testing and interpretation algorithms in some settings, supports the recommendation by the authors of using a more stringent IS*481* C_t_ cut-off (< 35) [16]. However, the 95% confidence intervals (28.5–38.1) imply that higher C_t_ values could still indicate positivity and, in a setting where testing procedures are standardised, should not be ignored. Also, C_t_ values in clinical specimens may vary due to specimen composition or may be affected during storage or transit, and thus may not directly emulate experimental sensitivity in a controlled laboratory environment.

Sequencing confirmed the presence of the IS*481* gene from a subset of PCR-positive samples, ruling out non-specific amplification, primer dimer formation and probe degradation, however, one *B. pertussis* confirmed, and eight possible samples could not be confirmed by sequencing likely due to insufficient DNA concentration for Sanger sequencing. Although only 7% of possible *B. pertussis* cases tested positive for the *ptxS1* gene due to the lower sensitivity of the assay compared to IS*481*, we excluded *B*. *holmesii* from all IS*481* PCR-positive samples, suggesting that these were positive for *B. pertussis*.

Earlier studies have indicated that NP is the preferred specimen type for laboratory detection of *B. pertussis* [10, 33, 34]. The use of sputum for *B. pertussis* detection has been suggested by WHO [34], however, supporting data are lacking. In our study, testing of induced sputum in addition to NP samples increased the diagnostic yield of *B. pertussis* 5-fold among hospitalised SRI patients. This is similar to another South African study where the *B*. *pertussis* detection rate was 3.7% when testing NP specimens and increased to 7% when induced sputum specimens were additionally included [35].

The World Health Organization has recommended that all countries perform pertussis surveillance and have suggested that for resource-limited settings it can be linked to surveillance for other respiratory illnesses. Due to the lack of pertussis data in South Africa, our aim was to establish pertussis surveillance by linking it to an already established syndromic SRI and ILI surveillance platform. While not pertussis-specific surveillance, this enabled us to obtain pertussis data for the country at a low cost by leveraging systems and staff already in place. An additional benefit of this surveillance is that children and adults with mild and severe respiratory illness that do not necessarily present with typical pertussis symptoms were included, adding to our understanding of pertussis disease in individuals that are not clinically suspected to have pertussis. However, use of syndromic surveillance platforms for pertussis surveillance also has several drawbacks, which need to be acknowledged. As the case definition for ILI cases and SRI adult cases included fever, it is likely that some pertussis cases were missed, and the data likely represents an underestimation of disease prevalence. In addition, the interpretation and clinical relevance of PCR-positive individuals that do not present with typical pertussis symptoms is not well understood. However, despite these limitations, we detected *B. pertussis* by PCR in 1.1% of patients with either mild or severe respiratory illness, which correlates with another South African study that enrolled an infant cohort from 2012 to 2014 as part of the Drakenstein Child Health Study in the Western Cape and found an overall pertussis prevalence of 2% among infants presenting with pneumonia [36].

The study has limitations that need to be considered. The surveillance case definition was not specific for pertussis therefore the time to swabbing following disease onset could not be determined. Overall, there was a small number of pertussis cases, which limited our statistical power for analyses, and as a result, differences between the characteristics of confirmed and possible cases which may exist, may not have been detected. Pertussis vaccination history was not available for adults, and for children < 5 years of age the collected data were poor, so the impact of vaccination on pertussis disease and C_t_ value could not be determined.

In a controlled setting with standardised procedures and when contamination can conclusively be excluded, we suggest that IS*481*-positive specimens with C_t_ values < 40 should be considered indicative of *B. pertussis* infection. Low IS*481* C_t_ values (< 35) are more likely to represent true cases of pertussis disease, whereas specimens with high C_t_ values may represent *B. pertussis* carriage (as *B. pertussis* was detected in controls with higher C_t_ values), residual *B. pertussis* DNA following disease, or active disease especially in older individuals who may present atypically. As such, in a respiratory surveillance setting and for public health reporting, low bacterial loads may not reflect true cases of disease. However, for individual patient management, possible cases should be interpreted in the context of other clinical and epidemiological factors including patient symptoms, age, immune status and epidemiological links with laboratory-confirmed pertussis cases. *B. pertussis* carriage is not well understood, and further research is required to better understand the significance of PCR detection of *B. pertussis* in asymptomatic individuals and individuals lacking typical pertussis symptoms.

## Conclusion

In conclusion, based on the results presented in this manuscript, we believe that in the absence of a pertussis-specific surveillance, leveraging of an already existing respiratory illness surveillance system as recommended by the WHO will allow countries to monitor the trend of infection over time, detect outbreaks and allow further research into cases that present with atypical pertussis symptoms.

## Additional files

Additional file 1: Comparison of confirmed (*N* = 38) and possible (*N* = 22) pertussis cases (real-time PCR positive for IS*481* in nasopharyngeal specimens) in hospitalized patients with severe respiratory illness, South Africa, June 2012 – May 2016 (*N* = 60). (DOCX 18 kb)

Additional file 2: Comparison of confirmed (N = 32) and probable (*N* = 15) pertussis cases (real-time PCR positive for IS*481* in nasopharyngeal specimens) in patients with influenza-like illness, South Africa, June 2012 – May 2016 (N = 47). (DOCX 17 kb)

## Abbreviations

AF: Attributable Fraction
BLAST: Basic Local Alignment Search Tool
CI: Confidence Interval
Ct: Cycle threshold
ILI: Influenza-Like Illness
KTHC: Klerksdorp-Tshepong Hospital Complex
MALDI-TOF: Matrix-Assisted Laser Desorption/Ionization Time of Flight mass spectrometry
NICD: National Institute for Communicable Diseases
NP: Nasopharyngeal
OP: Oropharyngeal
PCR: Polymerase Chain Reaction
RNaseP: Human ribonuclease P
SRI: Severe Respiratory Illness
UTM: Universal Transport Media
WHO: World Health Organisation
