# Supplementary material for: Challenges and clinical relevance of molecular detection of Bordetella pertussis in South Africa
Source: BMC Infect Dis. 2019 Mar 21;19:276. doi: 10.1186/s12879-019-3869-7 (PMC6429695; doi:10.1186/s12879-019-3869-7)
Supplement: Supplementary file 1 — Comparison of confirmed (N = 38) and possible (N = 22) pertussis cases (real-time PCR positive for IS481 in nasopharyngeal specimens) in hospitalized patients with severe respiratory illness, South Africa, June 2012 – May 2016 (N = 60). (DOCX 18 kb) [file 12879_2019_3869_MOESM1_ESM.docx]

Additional file 1. Comparison of confirmed (N=38) and possible (N=22) pertussis cases (real-time PCR positive for IS*481* in nasopharyngeal specimens) in hospitalized patients with severe respiratory illness, South Africa, June 2012 – May 2016 (N=60)

| Characteristic | Confirmed pertussis^*^  n/N^#^ (%) | | Possible pertussis^*^  n/N^#^ (%) | OR^†^  (95% CI) | P value | |
| --- | --- | --- | --- | --- | --- | --- |
| Year |  |  |  |  |  |  |
| 2012 | 3/38 (8) | | 5/22 (23) | Reference |  | |
| 2013 | 7/38 (18) | | 3/22 (14) | 0.3 (0.04 – 1.8) | | 0.18 |
| 2014 | 10/38 (26) | | 5/22 (23) | 0.3 (0.05 – 1.8) | | 0.19 |
| 2015 | 17/38 (45) | | 8/22 (36) | 0.3 (0.05 – 1.5) | | 0.14 |
| 2016 | 1/38 (3) | | 1/22 (4.5) | 0.6 (0.03 – 13.6) | | 0.75 |
| Gender |  |  |  |  |  |  |
| Male | 17/38 (45) | | 9/22 (41) | Reference |  | |
| Female | 21/38 (55) | | 13/22 (59) | 1.2 (0.4 – 3.4) | 0.40 | |
| Age group^¶^ |  |  |  |  |  |  |
| <1 | 22/38 (58) | | 7/22 (32) | Reference |  | |
| 1-4 | 5/38 (13) | | 3/22(14) | 1.9 (0.4 – 9.2) | 0.42 | |
| 5-14 | 2/38 (5) | | 1/22 (4.5) | 1.8 (0.2 – 16.0) | 0.60 | |
| 15-24 | 0/38 (0) | | 1/22 (4.5) | 9.0 (0.3 – 245.4) | | 0.19 |
| 25-44 | 6/38 (16) | | 7/22 (32) | 3.5 (0.9 – 13.2) | | 0.07 |
| 45-64 | 2/38 (5) | | 3/22 (14) | 4.2 (0.7 – 26.0) | | 0.12 |
| ≥65 | 1/38 (3) | | 0/22 (0) | 1.0 (0.04 – 27.3) | | 1.0 |
| Fever history |  |  |  |  |  |  |
| No | 19/38 (50) | | 13/22 (59) | Reference |  | |
| Yes | 19/38 (50) | | 9/22 (41) | 0.7 (0.2 – 2.0) | 0.50 | |
| HIV status |  |  |  |  |  |  |
| Uninfected | 25/37 (68) | | 11/20 (55) | Reference |  | |
| Infected | 12/37 (32) | | 9/20 (45) | 1.7 (0.6 – 5.2) | 0.35 | |
| HIV treatment |  |  |  |  |  |  |
| No | 2/8 (25) | | 5/8 (62.5) | Reference |  | |
| Yes | 6/8 (75) | | 3/8 (37.5) | 0.2 (0.02 – 1.7) | 0.14 | |
| Symptom duration |  |  |  |  |  |  |
| <7 days | 27/38 (71) | | 13/22 (59) | Reference |  | |
| 7-20 days | 5/38 (13) | | 4/22 (18) | 1.6 (0.3 – 7.2) | 0.50 | |
| ≥21 days | 6/38 (16) | | 5/22 (23) | 1.7 (0.4 – 6.7) | 0.43 | |
| Underlying illness^‡^ |  |  |  |  |  |  |
| No | 32/38 (84) | | 19/22 (86) | Reference |  | |
| Yes | 6/38 (16) | | 3/22 (14) | 0.8 (0.2 – 3.8) | 0.82 | |
| ICU |  |  |  |  |  |  |
| No | 35/37 (95) | | 21/22 (95) | Reference |  | |
| Yes | 2/37 (5) | | 1/22 (5) | 0.8 (0.07 – 9.8) | 0.89 | |
| Antibiotic treatment  (24 hours) |  |  |  |  |  |  |
| No | 33/38 (87) | | 20/22 (91) | Reference |  | |
| Yes | 5/38 (13) | | 2/22 (9) | 0.7 (0.1 – 3.7) | 0.64 | |
| Hospital duration |  |  |  |  |  |  |
| <2 days | 4/37 (11) | | 2/20 (10) | Reference |  | |
| 2-4 days | 12/37 (32) | | 10/20 (50) | 1.7 (0.3 – 11.1) | 0.60 | |
| 5-7 days | 7/37 (19) | | 4/20 (20) | 1.1 (0.1 – 9.3) | 0.90 | |
| ≥8 days | 14/37 (38) | | 4/20 (20) | 0.6 (0.08 – 4.3) | 0.59 | |
| Viral co-infection |  |  |  |  |  |  |
| No | 18/38 (47) | | 13/22 (59) | Reference |  | |
| Yes | 20/38 (53) | | 9/22 (41) | 0.6 (0.2 – 1.8) | 0.38 | |
| Outcome^¶^ |  |  |  |  |  |  |
| Survived | 34/37 (92) | | 22/22 (100) | Reference |  | |
| Died | 3/37 (8) | | 0/22 (0) | 0.2 (0.01 – 4.4) | 0.11 | |
| Vaccination for age^§^ |  |  |  |  |  |  |
| Full coverage | 15/22 (68) | | 7/10 (70) | Reference |  | |
| Incomplete | 5/22 (23) | | 3/10 (30) | 0.8 (0.1 – 4.2) | 0.77 | |
| Facility |  |  |  |  |  |  |
| Edendale | 19/38 (50) | | 7/22 (32) | Reference |  | |
| KTHC | 19/38 (50) | | 15/22 (68) | 2.1 (0.7 – 6.4) | 0.71 | |

OR = Odds ratio; CI = Confidence interval. ^*^Confirmed case=positive for *B*. *pertussis* with IS*481* C_t_ <35; Possible case=positive for *B*. *pertussis* with IS*481* 35≥C_t_≤39. ^#^Data unknown/missing for some cases accounting for the different denominators. ^†^Odds ratio was calculated for confirmed versus possible pertussis cases using univariate logistic regression.

^‡^Patients with previously diagnosed chronic conditions including asthma, chronic lung diseases, cirrhosis/liver failure, chronic renal failure, heart failure, valvular heart disease, coronary heart disease, immunosuppressive therapy, splenectomy, diabetes, burns, kwashiorkor/marasmus, nephrotic syndrome, spinal cord injury, seizure disorder, emphysema, or cancer. ^§^For children ≤5 years of age where vaccine history was available and documented on vaccination card. All percentages are rounded off. ^¶^ Estimated using penalized logistic regression.
