# Supplementary material for: Challenges and clinical relevance of molecular detection of Bordetella pertussis in South Africa
Source: BMC Infect Dis. 2019 Mar 21;19:276. doi: 10.1186/s12879-019-3869-7 (PMC6429695; doi:10.1186/s12879-019-3869-7)
Supplement: Supplementary file 2 — Comparison of confirmed (N = 32) and probable (N = 15) pertussis cases (real-time PCR positive for IS481 in nasopharyngeal specimens) in patients with influenza-like illness, South Africa, June 2012 – May 2016 (N = 47). (DOCX 17 kb) [file 12879_2019_3869_MOESM2_ESM.docx]

Additional file 2: Comparison of confirmed (N=32) and probable (N=15) pertussis cases (real-time PCR positive for IS*481* in nasopharyngeal specimens) in patients with influenza-like illness, South Africa, June 2012 – May 2016 (N=47).

| Characteristic | Confirmed pertussis^*^  n/N^#^ (%) | | Possible pertussis^*^  n/N^#^ (%) | OR^†^  (95% CI) | P value | | |
| --- | --- | --- | --- | --- | --- | --- | --- |
| Year |  |  |  |  |  |  |  |
| 2012 | 3/32 (9) | | 4/13 (31) | Reference |  | | |
| 2013 | 3/32 (9) | | 0/13 (0) | 0.1 (0.004 – 2.9) | | 0.19 | |
| 2014 | 8/32 (25) | | 4/13 (31) | 0.6 (0.1 – 3.4) | | 0.56 | |
| 2015 | 17/32 (53) | | 4/13 (31) | 0.2 (0.04 – 1.1) | | 0.07 | |
| 2016 | 1/32 (3) | | 1/13 (8) | 0.8 (0.05 – 11.1) | | 0.85 | |
| Gender |  |  |  |  |  |  |  |
| Male | 10/32 (31) | | 4/14 (29) | Reference |  | | |
| Female | 22/32 (69) | | 10/14 (71) | 1.1 (0.3 – 4.5) | 0.89 | | |
| Age group^¶^ |  |  |  |  |  |  |  |
| <1 | 4/32 (12.5) | | 0/15 (0) | Reference |  | | |
| 1-4 | 3/32 (9) | | 6/15 (40) | 16.7 (0.7 – 409.1) | | | 0.08 |
| 5-14 | 7/32 (22) | | 2/15 (13) | 3.0 (0.1 – 77.6) | | | 0.51 |
| 15-24 | 4/32 (12.5) | | 5/15 (33) | 11.0 (0.5 – 263.5) | | | 0.14 |
| 25-44 | 11/32 (34) | | 1/15 (7) | 1.2 (0.04 – 34.5) | | 0.93 | |
| 45-64 | 3/32 (9) | | 1/15 (7) | 3.9 (0.1 – 126.7) | | 0.45 | |
| Fever history |  |  |  |  |  |  |  |
| No | 1/32 (3) | | 2/14 (14) | Reference |  | | |
| Yes | 31/32 (97) | | 12/14 (86) | 0.2 (0.02 – 2.3) | 0.02 | | |
| HIV status |  |  |  |  |  |  |  |
| Uninfected | 15/29 (52) | | 6/13 (46) | Reference |  | | |
| Infected | 14/29 (48) | | 7/13 (54) | 1.3 (0.3 – 4.6) | 0.74 | | |
| HIV treatment |  |  |  |  |  |  |  |
| No | 4/13 (31) | | 2/7 (29) | Reference |  | | |
| Yes | 9/13 (69) | | 5/7 (71) | 1.1 (0.1 – 8.4) | 0.92 | | |
| Symptom duration |  |  |  |  |  |  |  |
| <7 days | 24/31 (77) | | 9/12 (75) | Reference |  | | |
| 7-20 days | 7/31 (23) | | 2/12 (17) | 0.9 (0.2 – 4.3) | | 0.85 | |
| ≥21 days | 0/31 (0) | | 1/12 (8) | 7.7 (0.3 – 207.1) | | 0.22 | |
| Underlying illness^‡^ |  |  |  |  |  |  |  |
| No | 31/32 (97) | | 14/14 (100) | Reference | |  | |
| Yes | 1/32 (3) | | 0/14 (0) | 0.7 (0.03 – 18.9) | | 0.85 | |
| Vaccination for age^§^ |  |  |  |  |  |  |  |
| Full coverage | 4/6 (67) | | 2/3 (67) | Reference | |  | |
| Incomplete | 2/6 (33) | | 1/3 (33) | 1.0 (0.05 – 18.9) | | 0.57 | |
| Facility |  |  |  |  |  |  |  |
| Edendale | 21/32 (66) | | 7/15 (47) | Reference |  | | |
| Jouberton | 11/32 (34) | | 8/15 (53) | 0.5 (0.1 – 1.6) | 0.22 | | |

OR = Odds ratio; CI = Confidence interval. ^*^Confirmed case=positive for *B*. *pertussis* with IS*481* C_t_ <35; Possible case=positive for *B*. *pertussis* with IS*481* 35≥C_t_≤39. ^#^Data unknown/missing for some cases accounting for the different denominators. ^†^Odds ratio was calculated for confirmed versus possible pertussis cases using univariate logistic regression. ^‡^Patients with previously diagnosed chronic conditions including asthma, chronic lung diseases, cirrhosis/liver failure, chronic renal failure, heart failure, valvular heart disease, coronary heart disease, immunosuppressive therapy, splenectomy, diabetes, burns, kwashiorkor/marasmus, nephrotic syndrome, spinal cord injury, seizure disorder, emphysema, or cancer. ^§^For children ≤5 years of age where vaccine history was available and documented on vaccination card. All percentages are rounded off. ^¶^ Estimated using penalized logistic regression.
